# Profiling resilience: A latent profile analysis of German nurses' coping and resilience

**DOI:** 10.3389/frhs.2022.960100

**Published:** 2022-12-08

**Authors:** Ingo Klingenberg, Stefan Süß

**Affiliations:** Chair of Business Administration, in Particular Work, Human Resource Management and Organization Studies, Heinrich Heine University, Düsseldorf, Germany

**Keywords:** stress, individual resilience, healthcare, coping, nursing, latent profile analysis

## Abstract

**Introduction:**

Because of the shortage of nurses, it becomes crucial for organizations and health systems to keep nurses in their workforce. As individual resilience is positively associated with organizational commitment and negatively with mental disorders, it may reduce nurses' intention to leave the profession. Thus, individual resilience gained attention in research. Nevertheless, there is no common conceptualization of individual resilience in the literature. Rather, three prevalent understandings exist. Due to these multiple understandings, the role of coping in the context of resilience remains unclear. Against this background, the aim of this study is to analyze the relationship between nurses' resilience and coping based on a person-centered approach.

**Methods:**

This study presents a latent profile analysis based on a survey of 210 German nurses. The profiles were generated based on the Brief Resilience Scale and Brief COPE. The Perceived Workload of Nurses' Scale and sociodemographic data were considered as explanatory factors using nominal logistic regression. Further, the relation with possible consequences was tested by *χ*²-test using the Irritation Scale and KUT Commitment Measure.

**Results:**

The study identifies four different profiles of coping and resilience. The profiles “resistant” and “social-active” show rather low irritations and high organizational commitment. The “passive” profile has lower irritation scores than the “solitary” profile does, but the “passive” profile is associated with more irritation than the “resistant” or the “social-active” profile. Whereas the other profiles include characteristics of resilience, the “solitary” profile has a vulnerable nature. The analysis shows that more coordination and information problems, higher age, and not being in a leadership role are associated with a higher probability of belonging to the “solitary” profile. The chance of belonging to the “solitary” profile is significantly higher for women than for men, whereas women have a significantly lower chance of belonging to the “resistant” profile, compared to men.

**Conclusion:**

The analysis shows that the three prevalent understandings of resilience are appropriate but it also indicates that future scientific debate requires more precision in defining individual resilience. The study contributes to sharpening the definition of resilience as well as to understanding the link between coping and resilience.

## Introduction

Most health services around the world face a shortage of specialists which threatens the health services' functionality (1). Especially nurses are in scarce supply in the labor market (2). This shortage is due to the low motivation of young people to be trained as nurses, nurses leaving the profession through early retirement—especially due to mental disorders—, and nurses changing professions (3). The reasons to leave the profession can be explained, amongst other things, by the high number of stressors in the daily work of nurses, such as workload, time pressure, aggression, as well as exposure to illness, injury, and death (4–7). Therefore, reducing stressors becomes a central task for health services to stay functional. However, as a lot of stressors result from nursing shortages (e.g., higher workload) or cannot be changed (e.g., patient deaths) the ability to withstand these stressors—individual resilience—is moving into the focus of research.

In general research, individual resilience is well investigated by stress researchers, who have reached consensus that individual resilience has positive effects for individuals and organizations. For instance, employees’ health (8) and organizational commitment (9) are positively associated with resilience. Therefore, individual resilience must be seen as part of organizations' path to establishing long-term relationships with their employees in terms of limiting absenteeism that is due to psychological or emotional issues and turnover intentions that are due to lack of commitment.

In particular, the resilience of nurses has attracted much attention in research. Several positive effects of nurses' individual resilience were found, as resilience can raise workability (10–13), foster empathy—an important ability in nursing—(14), and increase work satisfaction (15). Further, resilience can reduce the risk of leaving the profession (16, 17), and getting burnout (18).

Besides the effects of individual resilience, specifically the resilience of nurses has gained attention in research. As to whether nurses' resilience is associated with a specific sex, age range, or amount of work experience (19, 20), findings are mixed. Factors that have been shown to be positively associated or to positively affect nurses' resilience are emotional intelligence (21, 22), hope (10, 12), humor (23), a sense of coherence (24), social support (25–28), and religiosity (29, 30). Further, genetics, epigenetics, the developmental environment, psychosocial factors, neurochemicals, and functional neural circuitry can positively affect the development of nurses' resilience (31).

Whereas the literature offers plenty of empirical research, conceptual clearness is lacking. Although the literature often defines individual resilience as the ability to withstand adversity and stress and/or bounce back after a failure (31, 32), how individual resilience is conceptualized in the literature varies. Three conceptualizations of the concept are predominant: resilience as “resistance,” such that individuals are immune to strain; resilience as “regeneration,” such that an individual can recover from strain quickly, and resilience as “reconfiguration,” such that an individual can adapt to stressful environments (33).

These diverse conceptualizations of individual resilience are problematic in part because they limit the comparability of study results. As the broad range of definitions refer to resilience's intervening at various points in the stress process, the mechanisms behind resilience and its functionalities differ in scholars' understanding. In addition, the diverse definitions result in a lack of precise separation from other constructs, especially for the coping construct, which is sometimes described as a part of resilience and sometimes as a substitute for resilience (34, 35). Although it is doubtful that many scholars would deny that coping and resilience are related constructs (35), there is no consensus about the relationship between them. The lack of precision about the resilience construct also makes it impossible to establish a more systematic set-up of the construct in the resilience literature and could be a missing part of the theory-building puzzle.

To get more precision in how resilience should be defined, more research about how to characterize resilient individuals is necessary. The role of coping efforts may be a particularly useful indication of which definition of resilience best describes the phenomenon. The missing link between resilience and coping is often criticized in the literature (34–36); whereas this gap is sometimes addressed in conceptual papers, few empirical studies can be found. As a proper definition needs both a solid theoretical background and empirical evidence, further research is necessary. An empirical, person-centered approach can shed light on how constructs are related. Person-centered approaches put the subject into focus to explain the variance in a certain criterion. As (sub)populations, such as social groups share common characteristics and are homogenous internally while being heterogeneous to other groups. Typologies can be made following this logic based on person-centered approaches (37). A latent profile analysis (LPA) can be used to analyze data in a person-centered way. LPA approaches are already conducted in coping research (38–41). However, to the best of our knowledge, a person-centered approach that considers resilience and coping is missing.

Against this background, the aim of our study is to analyze the relationship between nurses' resilience and coping based on a person-centered approach. We expect our study to contribute to preventing shortages of nurses by improving the precision of the definition of individual resilience, adding to the scientific debate about the relationship between coping and resilience, and providing a typology that may be an innovation for the research on resilience and coping in nursing professions.

The article is structured as follows: The next section explains the concepts that are most relevant to this research, explains the terms stress and coping using a transactional model of stress, discusses the term individual resilience along its several definitions, and integrates it into the Transactional Model of Stress. The next section also offers four research questions that help to structure the analysis, while the third section explains our research design in terms of the methods used in data collection and data analysis and the scales and measures used. The fourth section presents the results and discusses them with regard to the research questions. The fifth and final section summarizes the study's contributions and limitations of our study and provides suggestions for future research.

## Conceptual background and research questions

### Transactional Model of Stress

By definition, adversity precedes resilience. A common example of adversity in the workplace is work-related stress (42). Our understanding of stress follows Lazarus and Folkman's (43) Transactional Model of Stress, which defines the starting point in the stress process as a stressor, that is, a stimulus that results from an environment and has the potential to cause a stress reaction. A stressor does not automatically lead to a stress reaction, as the reaction to a stressor is an individual matter. In the Transactional Model of Stress, appraisal and coping are also elements in the stress process.

According to Lazarus and Folkman (43), the stress process features two kinds of appraisal: primary and secondary. In the primary appraisal the individual (mostly unconsciously) appraises whether a stressor is positive, irrelevant, or dangerous and, if it is so appraised, then appraises whether the stressor is a challenge, a threat, or a harm/loss. Whereas a challenge is associated with a positive strain (comparable to eustress), a threat of something that may occur, and a harm/loss that has already occurred lead to a stress reaction. In the secondary appraisal, the individual judges (again mostly unconsciously) her or his ability to cope with the situation, and, if the individual has inadequate resources to cope, a stress reaction begins. According to Lazarus and Folkman, the primary and secondary appraisals are not independent of each other but are reciprocal. Both appraisals are influenced mainly by individual values and beliefs that, if misaligned with the environmental setting in the form of stressors, result in an individual's appraising a stressor as harm or loss.

If primary and secondary appraisals lead to a stress reaction, the human body is under tension. In particular, an exchange between the vegetative nervous system and adrenal cortex that leads to the production of the stress hormone cortisol and increases metabolism makes individuals more productive in the short run; however, in the long run, prolonged or chronic stress reactions can cause such dangerous health problems as a psychological disorder and cardiovascular disease (44).

The duration and intensity of a stress reaction depend mainly on the individual's coping strategies. Lazarus and Folkman (43) differentiate between two—or, in newer versions, among three—higher-order ways of coping. Problem-focused coping describes actions that aim to terminate the stressor, and emotion-focused coping strategies aim to regulate the emotions caused by a stress reaction. In newer versions of the Transactional Model of Stress, meaning-focused coping, which is often named as a third way to classify coping (45, 46), refers to coping strategies that affect individuals’ values and norms. The three kinds of coping strategies do not conflict; in fact, an individual is likely to combine them. Although coping aims to reduce a stress reaction, it can be functional or dysfunctional, such that it can reduce stress, have no effect, or even increase stress by creating new stressors or worsening negative emotions (35). Although the literature suggests that, for instance, social coping, active coping, and problem-focused coping are effective ways to cope with stress outcomes (47, 48), such may not always be the case. The goodness-of-fit hypothesis proposes that certain coping strategies fit certain situations but not others (33).

**Figure 1:**
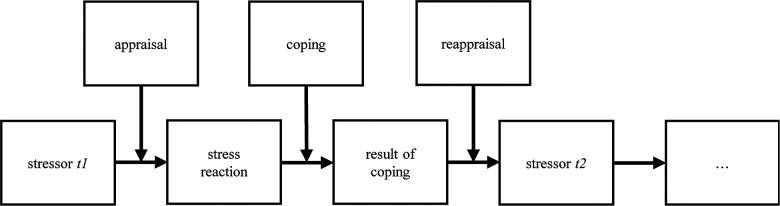
Transactional Model of Stress (following Lazarus/Folkman 1984).

A stress reaction lasts as long as the individual finds no way to cope or is no longer affected by the stressor. The last step in the Transactional Model of Stress is reappraisal, which describes the individual's evaluation of his or her coping's success. Finding a way to cope might influence an individual's future appraisals and tendency to evaluate a previous threat as a challenge (or vice versa). Figure 1 visualizes the stress process according to the Transactional Model of Stress.

The boxes show the steps in the stress process. The arrows show the direction of the process and the points at which appraisal, coping, and reappraisal intersect. The figure is constructed to be processual, so it holds for one stressor (t1) but can be repeated any number of times (tn). The “appraisal” box contains both primary and secondary appraisals.

### Individual resilience and its role in the stress process

Most scholars are likely to agree on a definition of individual resilience as the ability to withstand adversity/stress and/or bounce back after a failure (31, 32). However, this definition is vague, leaving how resilience affects the individual stress process unclear.

Three conceptualizations of resilience have been predominant in the scientific literature (33). The first conceptualizes resilience as “resistance,” (a) as being immune to strain. This conceptualization takes place at the (primary) appraisal level, such that individuals tend to appraise stressors as positive or irrelevant or as challenges that go along with eustress. The second conceptualization understands resilience as “regeneration,” (b) as being able to recover from strain rapidly, so resilience takes place at the coping level. Following this conceptualization of resilience, resilient individuals find functional strategies to cope with stress and end stress reactions more quickly than individuals who have less resilience do. Finally, resilience can be conceptualized as “reconfiguration” (c), meaning adapting to a stressful environment. This conceptualization takes place at the reappraisal level and tends to lead to positive (or at least less negative) future appraisals.

**Figure 2:**
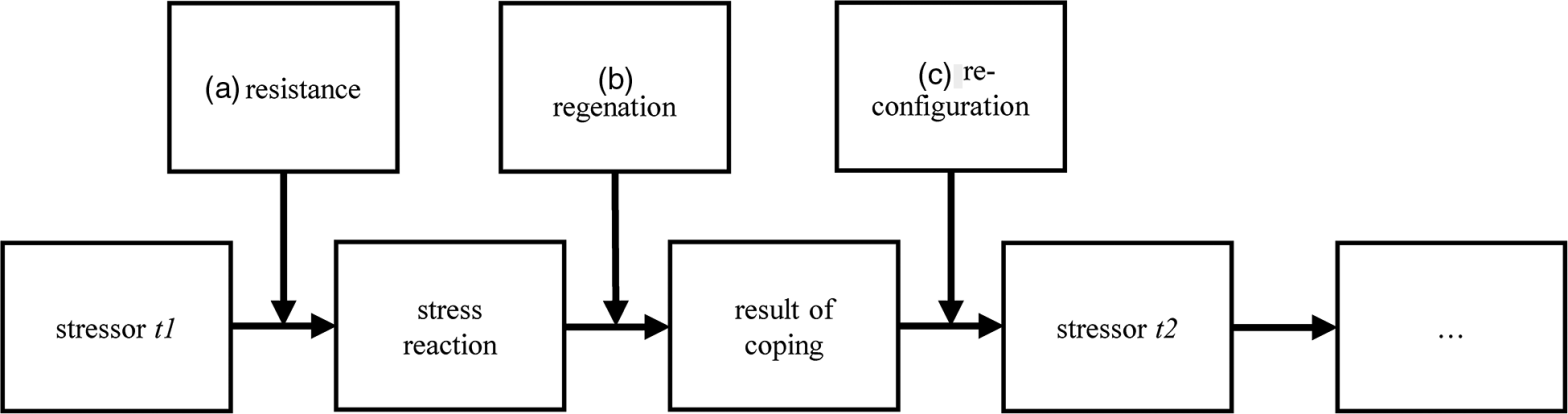
Incorporated Transactional Model of Stress and Resilience.

The lack of a common definition of individual resilience becomes visual when individual resilience is incorporated into the Transactional Model of Stress. Figure 2 takes up the stress process, as described in 2.1, and shows how the three conceptualizations of resilience can be incorporated into the stress process. The figure makes clear that the three conceptualizations’ mechanisms influence the stress process differently and affect the stress process at different stages.

### Development of research questions

Our study uses an explorative design, so no hypotheses regarding the composition of the profiles that will emerge from the LPA are made. Instead, the study is structured in terms of research questions.

Where resilience is placed in the stress process depends on its definition (Section 2.2). To refine its definition, it is necessary to analyze the ability of individuals to withstand stress and/or bounce back after a failure and to understand what coping looks like. By mapping the combination of coping and resilience into profiles, we aim to better understand in which way persons with varying degrees of individual resilience cope with stress. The advantage of a person-centered approach is that combinations of variables are simultaneously considered within different individuals. Therefore, a person-centered approach takes (possible) interactions of coping and resilience on the individual level into account. The goodness-of-fit hypothesis proposes no superior coping strategy, as different coping strategies can be more effective under different circumstances, and they can take place at the same time and can affect each other. Therefore, the profiles identified in the LPA should cover a broad range of ways of coping and resilience.

Consequently, the first research question is formulated as follows:

RQ1: What coping and resilience profiles can be identified?

The literature offers several factors to explain the occurrence of resilience, so these factors are also considered in our study. The literature suggests that individual factors and/or (successful) adaptation to stressors (33, 49) are the main drivers of resilience, so personal variables and stressors are taken into account in building the profiles. As adversity is determined by such environmental settings as stressors, we chose stressors that are typically part of nursing jobs. We also investigate whether coping and resilience profiles are determined by age, sex, or other job- and person-specific factors. Although our study design is explorative, we cannot take every possible explaining variable into account, so we forgo other individual characteristics known from research on resilience-building (see 1). Therefore, the second research question is:

RQ2: What factors can explain the differences in resilience profiles?

To understand how the profiles work, it is necessary to analyze their effects on associated variables. Individual resilience and effective coping have a negative effect on the incidence of mental disorder. Mental disorder is often a long-term consequence of long-term stress, so we investigate irritation as an earlier result of stress, a precursor of mental disorder, and a clear indication of incorrect workload (50, 51). Because of the current and ongoing shortage of nurses, we also consider organizational commitment as a possible factor in turnover intentions. Therefore, the third research question is:

RQ3: How do profiles differ with regard to the consequences of stress?

It is necessary for our discussion of the definition of resilience to address the combination of profiles, causes, and consequences. Therefore, a fourth, structure-related research question is issued for the conceptual discussion:

RQ4: How can profiles be explained based on the identified causes and consequences of stress?

## Materials & methods

### Data collection and sample

Data were collected between July and December 2019, so the results of the analysis must be seen in a pre-COVID-19 context. Caregiving jobs like nursing have experienced new settings and stressors during the pandemic, which began in Germany at the beginning of 2020, but nurses' stressors have always included heavy workloads, aggression, harm, and patient deaths (4–7). Therefore, the fact that the situation has worsened during the pandemic is not contrary to the conceptual discussion that is this study's focus.

Data collection was conducted using an online survey with a questionnaire of 105 items. The questionnaire was pre-tested for functionality and clarity of the items (*n* = 10). Participants had to answer a control question (Are you currently a nurse in the stationary device?) with “yes” beforehand, to participate in the survey. Data were collected cross-sectional, meaning we used a single time of measurement. The average time to complete the questionnaire was 16.6 min. We removed participants from the original sample (*n* = 47) if they answered the filter question with “no” (*n* = 20) or showed anomalies (*n* = 27) in their responses (e. g. implausible answers in age/duration of employment, statistical outliers, quick completion or a high tendency to answer in a social desirable way).

The sample consists of 210 participants. 156 (73.9%) are women and 55 (26.1%) are men. Participants range in age between 21 and 69 years (mean = 43.47; median = 43). With 93 (44%) participants each, the areas of geriatric nursing and healthcare nursing are evenly represented, while about 12 percent of participants work in other fields. Most participants work in private (43.1%), charitable (33.1%), or public organizations (21.3%), while about 11% work in other organizations. Comparing our data with official statistics (52) reveals that the sample has similarities to the German health service. However, due to its sample size, it cannot be considered representative.

Our sample consists of both examined nurses and nursing assistants. The majority belongs either to the group of examined geriatric nurses (*n* = 81, 38.4%) or examined healthcare nurses (*n* = 77, 36.5%). The remaining participants are geriatric nurses’ assistants (*n* = 12, 5.7%), healthcare nurse assistants (*n* = 16, 7.6%), and “others” (*n* = 25, 11.8%). The participants in the sample have different professional qualifications. Most participants (*n* = 74, 35.1%) have completed a vocational training. Many participants have either a general university entrance qualification (*n* = 50, 35.1%) or a secondary school certificate (*n* = 48, 22.7%). 28 participants (13.3%) have a university/technical college degree. Seven participants (3.3%) have a school leaving certificate. The other four participants have other forms of professional qualifications (e. g. foreign or outdated graduations).

### Data analysis

#### Factor analysis

An LPA requires a solid factor structure, so we performed a factor analysis in two steps. First, as the Brief COPE, which is often used to measure coping, may vary in terms of the number of factors (Section 3.3), we used an explorative factor analysis (EFA) to determine the coping factors. In the second step, we tested all factors using confirmatory factor analysis (CFA), which takes the chi-square (*χ*²) test, the Comparative Fit Index (CFI), the Tucker-Lewis-Index (TLI), the Standardized Root Mean Square Residual (SRMR), and the Root Mean Square Error of Approximation (RMSEA) into account.

#### LPA

We conducted a series of LPAs based on the Maximum Likelihood Estimate (MLR) for 1 to 5 profiles that consist of coping and resilience dimensions (Section 2.3). We took several criteria into account to evaluate the different numbers of profiles: The values of the Akaike Information Criterion (AIC) and Bayesian Information Criterion (BIC) should be low (53) and the entropy values should exceed 0.60 to reflect a good classification (54). Moreover, we used the (Vuong-) Lo-Mendell-Rubin Likelihood Ratio Test (LMRT) and a Parametric Bootstrapped Likelihood Ratio Test (BLRT) to contrast the probability of the number of profiles to a solution with fewer profiles. Finally, we used theoretical plausibly as perhaps the most useful criterion for building classes (55).

#### Nominal logistic regression (R3step)

Once we had determined the number of profiles, we tested possible explaining factors using nominal logistic regression (R3step in Mplus). The odds-ratio indicates whether a profile can be explained by certain variables. As explaining variables, we used several kinds of stressors from nursing jobs, along with the participants' sex, age, and function (leading function or not).

#### *Χ*²-test (BCH)

To test whether the profiles' influences regarding the outcomes of strain differ, we conducted a *χ*²-test (BCH in Mplus) using irritation, which is a precursor of mental disorder, and organizational commitment, which is related to stress.

### Operationalization

#### Stressors

We measured stressors using Perceived Workload of Nurses' Scale in German (56; *α* = 0.83). This scale, which was developed to examine stressors in nursing jobs, consists of twelve items that are measured with a five-point Likert scale (1 = very seldom—5 = very often). The scale contains two subscales, Coordination and Information Problems (exemplary item: “How often does it happen that you have to abide by regulations?”) and Psychophysical Overload (exemplary item: “How often does it happen that you feel completely exhausted after work?”). The scale was developed using three random samples in different healthcare settings. The initial number of considered items was halved from 24 to 12 items. Though no statistical information about the statistical validation was given by the authors (56), we considered the scale to be appropriate because it captures the specific work context of nurses. Therefore, the scale is much more suitable than non-nurses-specific measurements.

#### Individual resilience

We measured individual resilience using the German version of the Brief Resilience Scale (57; *α* = .85), which is based on an English scale (32), and was validated for the German context on a population-based and representative German sample. In the German validation study, the scale correlated positively with positive coping strategies, optimism, and well-being, whereas it correlated negatively with mental health measures (57). The scale consists of six items measured with a five-point Likert scale (1 = I do not agree at all—5 = I totally agree) and follows the conceptualization of resilience as reconfiguration (exemplary item: “I tend to bounce back after adversity”).

#### Coping

We measured coping using the German version of the Brief COPE (58; *α* = 0.70–0.81), which is a reduced version of the COPE and contains 28 items measured with a four-point Likert scale (1 = totally disagree; 4 = totally agree). The scale showed a lack of internal consistency and item variance during and after its developement (59). This is why the consideration of the items of the Brief Cope in research varies regarding the number of items and their assignment to different factors (the number of factors considered varies from 4 to 14) (59). Hence, an EFA was conducted to determine the number of factors and the factors' structure to be considered in this study.

#### Irritation

The construct of irritation describes a state between mental fatigue and mental disorder. Irritation might result from stress reactions (50), which we measured to indicate the consequences of strain using Mohr and Rigotti' s (51) irritation scale (*α* = 0.81). The scale was validated in several studies. It shows for instance positive correlations with anxiety and emotional exhaustion and negative correlations with life satisfaction and job satisfaction (51). The scale consists of eight items on a seven-point Likert scale (1 = totally disagree—7 = totally agree). The irritation scale can be divided into two subscales: emotional irritation (example statement: “I get annoyed rapidly”) and cognitive irritation (example statement: “I have difficulty relaxing after work”).

#### Commitment

Commitment can be influenced by strain, so we added commitment to our analysis to test the effects of the respective stress-management profiles. We measured commitment using the German Version of the KUT Commitment Measure (60; *α* = 0.89–0.92), which consists of four items that measure commitment to a certain target on a five-point Likert scale (1 = not at all—5 = extremely) [example question: “How committed are you to (target)?”]. Our study uses the targets job, organization, and colleagues, so the scale has twelve items. The scale was validated within a panel study that addressed employees. The commitment targets in the validation study were the organization, the leader, and the team of the participants. Negative correlations were found, for instance, for the intention to quit and positive correlations were found for the identification with the respective commitment target (60).

We also asked the respondents to answer questions concerning their job characteristics and sociodemographic data and to respond to a scale for social desirability (61; *α* = 0.71–0.78). We used these factors for control purposes and as possible explanatory variables.

## Results

### Factor analyses

The EFA we conducted to determine, based on the Kaiser-criterion (Eigenvalue > 1), the number of coping factors led us to conclude that three factors of coping should be built. Following Knoll et al.'s (58) suggestion that the number of subscales be reduced from 14 to 11 because of a lack of internal consistency and item variance, we excluded several variables, including alcohol and drug use. Items with a low factor loading were also excluded. For instance, the dimensions of religiosity and venting that have low loadings on any factor, so they were excluded. The three factors we built are explained in the following sections.

#### Active coping

The active coping factor is based on three items, two of which are from the original dimension of “positive reframing,” while the third is from the dimension of “active coping.” All items have an active character, and coping strategies require a certain level of activity, so we use the term “active coping.” In contrast to Knoll et al.'s (58) factor structure, our factor structure does not differentiate between emotion- and problem-focused. The Cronbach's *α* value of the scale is 0.576, which is critical. A low Cronbach's *α* value might be an indication of a complex construct. This holds true for coping strategies, whose complexity is reflected in the ongoing debate about how to classify them adequately (62). Though low consistency might be a limitation, the EFA reveals the factor structure to be the best solution.

#### Social coping

The second factor is also based on three items, of which two use the dimension “use of instrumental support” and one uses the dimension “use of emotional support.” A similar factor can be found in Knoll et al.'s (58) suggested structure, but our built structure consists of fewer items. Nevertheless, the factor is comprehensive, as it comprises coping strategies that use the help of others. The Cronbach's *α* value of the scale (0.815) can be considered good.

#### Evasive coping

The third factor also consists of three items, two from the “self-blame” dimension and one from the “denial” dimension. As both strategies are ways of circumventing problems, we chose the term “evasive coping,” which is comparable to Knoll et al.'s (58) factor of the same name. That we found no coping strategy from the “venting” dimension might be because of the settings in which nursing took place, which may not allow for venting without disturbing patients. The Cronbach's *α* value of the scale is 0.643 and can be considered questionable.

The CFA we conducted to test the factor structure of the newly built variables and the factors of the other variables shows an acceptable structure (*χ*² (924) = 1.243; *p* > 0.000; CFI = 0.916; TLI = 0.906; RMSEA = 0.04 (90% CI: 0.034–0.046); SMR = 0.056). As with the EFA, we excluded items with low factor loadings. The Cronbach's *α* values of the scales we used, coordination and information problems (0.793), psychophysical overload (0.789), resilience (0.72), Emotional Irritation (0.795) Cognitive Irritation (0.875), and commitment to the job (0.806) commitment to the Organization (0.882), and commitment to colleagues can be considered good to acceptable. LPA assumes local independence and normal distributions of the outcome variables within each class (63). Since all variables were uncorrelated and the Skewness/Kurtosis scores had values between +1 and −1, we considered the conditions of normal distribution and local independence to be met. Table 1 shows the correlation matrix of the variables used.

**Table 1:** Correlation matrix (75).

	STRESS1	STRESS2	Res	COPE1	COPE2	COPE3	IRR1	IRR2	COMJ	COMO	COMC	Age	Sex	LF	LE	Inter	CP	TT	subA	WE	subE	SoA
STRESS1	1																					
STRESS2	,636**	1																				
Res	−,484**	−,355**	1																			
COPE1	−0,012	−,160*	0,029	1																		
COPE2	−0,075	−,140*	,292**	,222**	1																	
COPE3	,266**	,207**	−,278**	0,106	0,131	1																
IRR1	,556**	,575**	−,489**	−0,076	−,211**	,300**	1															
IRR2	,435**	,399**	−,351**	0,007	−0,094	,323**	,530**	1														
COMJ	−,195**	−,262**	,235**	,142*	,194**	−0,010	−,233**	0,033	1													
COMO	−,367**	−,452**	,261**	,233**	,154*	−0,082	−,316**	−0,129	,521**	1												
COMC	−,205**	−,353**	,194**	,309**	0,085	−0,009	−,257**	−0,016	,544**	,470**	1											
Age	−0,096	−,168*	0,080	0,011	0,028	0,093	−0,081	0,034	,172*	,176*	,276**	1										
Sex	0,089	0,011	−0,081	0,044	0,040	0,095	0,105	0,090	0,081	0,058	,161*	0,030	1									
LF	,259**	,297**	−,258**	0,007	−0,086	0,118	,172*	−0,001	−,308**	−,432**	−,240**	−,186**	0,042	1								
LE	0,089	,143*	−0,063	0,050	0,004	0,121	,145*	−0,031	−0,087	−0,113	−0,116	−0,130	0,043	0,109	1							
Inter	,408**	,399**	−0,127	−0,084	−0,100	0,078	,255**	,252**	−0,083	−,254**	−0,016	−0,078	0,009	−0,082	0,049	1						
CP	0,058	0,030	0,010	−0,068	0,033	−0,007	−0,008	−0,074	0,075	−0,020	0,003	−0,129	0,126	,172*	0,110	0,095	1					
TT	−,479**	−,412**	,159*	,151*	,182**	−,172*	−,234**	−,230**	0,045	,322**	0,086	0,049	0,011	−,265**	−0,087	−,402**	−0,021	1				
subA	−,362**	−,435**	,298**	,190**	,179**	−,136*	−,319**	−,207**	,315**	,385**	,249**	0,084	0,038	−,176*	−0,116	−,294**	0,102	,301**	1			
We	,434**	,381**	−0,094	0,027	−0,062	,182**	,295**	,224**	0,009	−0,103	−0,083	−0,003	0,039	,208**	0,122	,276**	0,061	−,329**	−0,129			
subE	,491**	,317**	−,210**	0,029	−0,017	,169*	,288**	0,119	−,151*	−,266**	−0,066	−0,034	0,073	,229**	,149*	,343**	0,124	−,420**	−0,134	,343**	1	
soA	,395**	,484**	−,151*	0,041	−0,028	0,086	,318**	,152*	−,192**	−,286**	−,178**	−0,132	−0,022	0,125	,153*	,407**	0,006	−,336**	−,294**	,351**	,394**	1

* Correlation is significant at the level of 0,05.

** Correlation is significant at the level of 0,01.

STRESS1, coordination and information problems; STRESS2, psychophysical overload; Res, resilience; COPE1, active coping; COPE2, social coping; COPE3, evasive coping; IRR1, emotional irritation; IRR2, cognitive irritation; COMJ, commitment to job; COMO, commitment to organisation; COMC, commitment to colleauges; LF, leading function; LE, limited employment; Inter, interruptions during work; CP, contact to patients; TT, time for tasks; subA, appreciation; WE, working environment; subE, subjective efforts; soA, scope of actions;.

### Profiles

We conducted a series of LPAs (1–5 profiles), the results of which are shown in Table 2. Based on the BIC, the LMRT, and theoretical plausibility, we determined that the four-profile solution is most plausible. BIC reaches its lowest point and the LMRT has no significance in every solution with more profiles.

**Table 2:** Overview of fit values LPA (75).

#	LL	Par.	AIC	BIC	Entropy	BLRT	LMRT
1	1009,942	8	2035,884	2062,66	n/a	n/a	n/a
2	−988,598	13	2003,196	2046,708	0,787	42.688***	41.148***
3	−971,968	18	1979,937	2040,185	0,725	33.259***	32.060
**4**	−**954,294**	**23**	**1954,588**	**2031,571**	**0,724**	**35**.349***	**34**.075**
5	−945,327	28	1946,653	2040,372	0,737	17.934**	17.288

#, number profiles; LL, log likelihood; #Par., number parameter; AIC, akaike information criterion; BIC, bayesian information criterion; LMRT, vuong-lo-mendell-rubin likelihood ratio test; BLRT, parametric bootstrapped likelihood ratio test; **p < .05; *p < 0.01.

**Figure 3:**
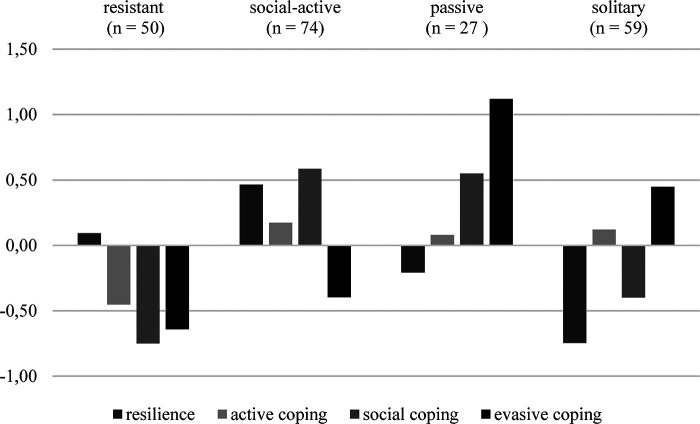
Overview of profiles (75).

Figure 3 shows the profiles. One participant was excluded after the first round of profile analysis because of an indication of statistical outliers. The numbers to the left represent the divergence between the overall sample's mean and the mean of the respective variables in the profiles. The names in the top of the figure are the names of the profiles. The bars show the direction and the amount of divergence between the full sample's and the profiles' means of the respective variables.

The name of the first profile (*n* = 50) is “resistant.” It is characterized by its (slightly) above average resilience and below average values in all three coping dimensions. We named the profile after one of the conceptualizations of resilience, as the profile shows high resilience values combined with low coping efforts. This combination might be interpreted as a profile that masters stress on the appraisal level.

The name of the second profile (*n* = 74) is “social-active” because it has two dominant characteristics: Above average resilience and above average social coping. The profile also shows slightly above average active coping and below average evasive coping.

The third profile (*n* = 27), named “passive,” has two dominant characteristics, as the dimensions of evasive coping and social coping are above average. Hence, the dominant strategies are those that do not take action. Active coping is only slightly above average, and resilience is below average.

The fourth profile (*n* = 59), named “solitary,” has four dominant characteristics: above average evasive coping, below average resilience, below average social coping, and above average active coping. The name “solitary” is appropriate because these individuals seem to prefer coping strategies that do not require social support.

### Explanatory and dependent variables

To expand our interpretation of the profiles, we also examined causes and effects. As possible causes, we took into account two categories of stressors: coordination and information problems and psychophysical overload, along with age, sex, and having a leading function. For consequences, we examined cognitive and emotional irritations, as well as commitment to the job, the organization, and colleagues.

We analyzed explanatory factors using multinomial logistic regression analysis, so we calculated the odds ratio (OR) to determine the chance of belonging to a particular profile and tested it for significance. Table 3 shows the calculated coefficients, the standard error, the OR, and the significance in the higher chance in the between profile comparison. The analysis shows that more coordination and information problems, higher age, and not being in a leading role go along with a higher probability of belonging to the “solitary” profile. The chance of belonging to the “solitary” profile is significantly higher for women than is belonging to the “resistant” profile. No other comparisons show significant differences.

**Table 3:** Overview of odds ratio with regard to explaining variables (75).

	Profile 1 vs. 2		Profile 4 vs. 2		Profile 3 vs. 2		Profile 4 vs. 1		Profile 3 vs. 1		Profile 3 vs. 4	
	Coef. (SE)	OR	Coef. (SE)	OR	Coef. (SE)	OR	Coef. (SE)	OR	Coef. (SE)	OR	Coef. (SE)	OR
Stress1	−0.455 (0.338)	0,634	**1.438** (0,806)*	**4,212**	0.064 (0.377)	1,066	**1.893 (0.753** **	**6,639**	0.519 (0.364)	1,680	−**1.374** (0.827)*	**0,253**
Stress2	0.354 (0.432)	1,425	0.537 (0.491)	1,711	0.204 (0.445)	1,226	0.184 (0.470)	1,202	−0,149 (0,470)	0,862	−0.333 (0.539)	0,717
sex	−0.721 (0.507)	0,486	1.114 (1.096)	3,047	−0.747 (0.619)	0,474	**1.835** (1.084)*	**6,265**	−0.026 (0.576)	0,974	−1.861 (1.208)	0,156
age	0.008 (0,023)	1,008	**0.126** (0.058)**	**1,134**	0.010 (0.028)	1,010	**0.119** (0.055)**	**1,126**	0.002 (0.027)	1,002	−**0.117** (0.066)*	**0,890**
leading function	0.483 (0,529)	1,621	**3.333** (1.471)**	**28,022**	−0,903 (0.973)	0,405	**2.850** (1,377)**	**17,288**	−1,386 (0,997)	0,250	−**4.235** (1.877)**	**0,014**

Coef., coeffcient; SE, standard error; OR, odds ratio.

** *p* < .05.

* *p* < 0.01; Profile 1 = „resistant“; Profile 2 = sozial-active; Profile 3 = „passive“; Profile 4 = „solitary“.

The effects of the profiles were analyzed using a *χ*²-test (BCH). Table 4 summarizes the means with regard to the respective dependent variable, as well as the significance comparisons between the profiles. The analysis shows that the “resistant” and “social-active” profiles are associated with the least amount of irritation, while the “solitary” profile is associated with the highest irritation scores. The “passive” profile has lower irritation scores than the “solitary” profile does, but the “passive” profile is associated with more irritation than the “resistant” or “social-active” profiles. As for commitment to the job, the “solitary” profile has significantly lower values than any other profile, and the “social-active” profile has significantly higher scores than the “resistant” profile does. Concerning commitment to the organization, the “solitary” profile has significantly lower values than the “passive” and “social-active” profiles do. All profiles have similar results for commitment to colleagues, with significant differences only between the highest value in the “social-active” profile and the lowest value in the “solitary” profile.

**Table 4:** Overview of means of dependent variables (75).

	Profile 1	Profile 2	Profile 3	Profile 4	
	Resistant	Social-active	*P*assive	Solitary	Significantly diffence
Cognitive irritation	2,357	2,051	2,739	3,300	1 = 2; 1 = 3; 3 > 2; 1,3, < 4
Emotional irritation	2,860	3,109	3,829	4,542	1 = 2, 1 < 4, 3; 3 = 4, 1, 2 < 4
Commitment job	4,469	4,706	4,684	4,250	4 < 1,2,3
Commitment organization	3,939	4,219	4,114	3,595	4 < 2,3; 1 < 2
Commitment collequges	4,093	4,329	4,235	4,042	4 < 2

## Discussion

### Discussion of the results

The first research question of our study addressed the question which profiles of coping and resilience can be identified. The analysis shows that a solution with four profiles is best. The profiles are described in 4.2. We found profiles that differ in the combinations and the extent of coping and resilience. To give meaning to in what way these profiles represent resilience, we need more information about the explaining factors and the consequences of the profiles.

The second research question of our study was about which factors can explain the identified profiles. The analysis shows that a higher amount of coordination and information problems, higher age, and not having a leadership role go along with a higher probability of belonging to the “solitary” profile. The chance of belonging to the “solitary” profile is significantly higher for women than for men, whereas women have a significantly lower chance of belonging to the “resistant” profile, compared to men..

Our third research question asked how the profiles differ with regard to the consequences of stress. The “resistant” and “social-active” profiles show comparatively low values of irritation and high values of organizational commitment. The “passive” profile has lower irritation scores than the “solitary” profile, but the “passive” profile is associated with more irritation than the “resistant” or “social-active” profile.

The fourth research question of our study asks how the profiles can be explained based on the identified causes and consequences of stress. The sum of causes, effects, and functioning can be used for a comprehensive interpretation of the profiles, as given in this section.

(1) The “resistant” profile is the representation of the resilience definition of resistance (see Section 2.2). The profile is characterized by above-average resilience and below-average coping efforts. Respondents assigned to this profile have lower irritation scores and higher commitment scores, but they do not face fewer stressors than other profiles. Therefore, those who belong to this group are able to regulate stress on the appraisal level. While our study reveals no significant causes for this, it is possible that other variables than those we included explain belonging to this profile. We contend that the characteristics that influence consciousness might affect resilience and suggest mindfulness as a factor. The higher awareness and attentiveness that go along with mindfulness (64, 65) might also go along with neutral appraisal.
(2) The “social-active” profile is characterized by above-average resilience and above-average use of coping strategies that the literature has found have positive effects on social and active coping (47, 48). The profile is also characterized by low irritation and high commitment. As the profile shows higher coping effort than the “resistant” profile does, we conclude that the stress process ends after coping such that the profile is in accordance with the resilience definition of regeneration. Our analysis reveals no ex-plaining factors, but we suggest that future research consider hope as a possibility. According to Lazarus (66, p. 674), hope is a “resource for coping”(p. 674), meaning that hope—apprehended as believing in a positive outcome—is necessary for individuals to apply coping strategies.
(3) The “passive” profile has a slightly below average amount of resilience and an above average amount of evasive coping, along with a medium level of irritation and commitment. Thus, the effects of stress are present, but so is a level of resilience. We conclude that this profile represents, to an extent, the definition of resilience as reconfiguration. Individuals who fit this profile adapt to their environment by accepting it and avoiding problems, a form of reconfiguration. Although this approach is not the most effective for organizations or individuals, it is helpful in withstanding a stressful environment. In nursing jobs, this kind of resilience might show up in individuals' abbreviating work processes, which is comparable to job-avoidance crafting (67, 68), that is, employee-sided recreation of work content (69). Although we found no evidence of explanatory factors, future research could determine whether factors that explain avoidance-job-crafting, such as cynicism (67), can be associated with this profile, after which a discussion about the comparability of the two constructs would be necessary.
(4) The “solitary” profile is characterized by below-average resilience and social coping. This group tends to be vulnerable to stress, as it has the highest irritation scores and the lowest commitment scores. These people are worth being protected by organizations, as they are most likely to leave their organizations or even their professions. The chance that one will belong to this group is highest for older women who do not have a leading position, although the study does not explain why this is the case. An explanation might be found in the theory of learned helplessness (70), where individuals do not find an adequate way of coping with problems and no longer try to change things.

Besides the conceptual discussion, our results have to be discussed with regard to the existing research on the resilience of nurses. The results are in line with research that reveals that resilience reduces the risks of leaving the profession (16, 17) and of developing mental disorders (18). Even resilience in form of a reconfiguration, with a passive way of coping, reduces the named risks. Our study reveals that being female and older is associated with lower levels of resilience. Because there have been inconsistent findings regarding the role of gender and age in the literature on resilience to date (19, 20), no general conclusions can be drawn from this. However, reviewing possible moderating and mediating factors might help explain the role of sex and age in future research. We suggest to investigate the role of (not) occupying a leadership position as a possible variable, which in our study increases the chance of belonging to the “solitary” profile. We further suggest giving more attention to the role of social support. Social support in research is often considered as a factor that fosters individual resilience (25–28). The “passive” profile indicates that social support may additionally go along with evasive coping.

### Contributions

Our analysis makes several contributions to the scientific literature and the nursing profession. First, we give new input into the discussion around the definition of resilience. Our discussion shows that individual resilience can be conceptualized in three ways. As research lives by the understanding of constructs that can be limited precisely and differentiated from other constructs, we ask the scientific community to scrutinize the definitions of resilience more closely. Our findings suggest differentiating among various kinds of resilience, and we argue that we should stick to the terms “resistance,” “regeneration,” and “reconfiguration.”

Second, our study contributes to the question concerning how coping and resilience are associated, which has often been addressed (34–36). Our results show that, depending on how resilience is interpreted, resilience might be a substitute for coping, part of coping, or a result of coping. To understand the construct properly, a clear definition is required and somewhat urgent.

Third, we contribute to the understanding of coping and resilience in the field of nursing. Our results show that women, older people, and people who do not hold a leading position in their organizations have a higher risk of belonging to a group that is vulnerable to stress. This finding contradicts in part literature outside the nursing sector that often views women and older peoples as more resilient than men and younger people are (71, 72). If there is an explanatory mechanism, such as a loss of resilience as a result of not achieving a leading function, or if this finding is a result of the sample structure (or the population that above average consists of women) must be determined in further research. Our study goes along with studies that have shown that active, adaptive and social coping are often associated with good outcomes (47, 48). However, our results suggest that the combination of coping strategies must be considered more thoroughly in future research, as people are likely to use several coping strategies at once, which might influence each other.

Fourth, our study offers methodological contributions to the field of resilience research. Person-centered studies in general and LPAs in particular are seldom found in the extant resilience research. Making the person the focus offers a new perspective for resilience research and contributes clarifying the mechanisms of individual resilience.

Fifth, our study also makes practical contributions. People in healthcare and nursing face several stressors (4–7), particularly now, when the shortage of specialists and the high demands that have resulted from the COVID-19 pandemic have increased stress in healthcare work even more, making the topic is highly relevant to healthcare organizations and the public health systems. Our study offers the perspective of the individual who must deal with (increasing) stress. The analysis shows that coping with stress can lead to behaviors that are not in line with organizational goals (as in the “passive” profile) or to lacking any adequate way to cope, as found in the “solitaire” profile. Neither would be in the interest of organizations. Therefore, we advise organizations to take the topic of stress seriously by promoting health-related resources and protecting employees from stressors wherever possible.

Finally, our study contributes to resilience research in general, as most fields must address the question concerning what makes individuals, organizations or systems resilient. Our discussion of the conceptualization of resilience can be transferred to areas other than nursing. Our typology offers an explanation for the concept's possible mechanisms. Like individuals, systems and organizations can show resistance, regeneration, or reconfiguration, so the question concerning what is meant by resilience should be taken more into account in other areas than health-services as well.

### Limitations and future research

Our study has some limitations. One common problem with survey studies is that findings are based on self-assessment by respondents, who may have retrospective biases or (current) situational factors that can influence their judgment of the past and/or the social desirability of their responses. The last of these holds especially for answers concerning mental health; problems in mental health are often hidden because of fear of stigmatization and prejudices by others (73). To avoid problems like self-assessment biases and answers based on social desirability, more objective measures should be considered for future research. Proxy judgments by others and (participatory) observations can offer an alternative to self-assessment, and such objective measures as blood pressure and other physical functions could be more taken into account. To reduce the effects of situational factors, diary studies could also be an option for gaining long-time insights into coping and resilience.

Other limitations result from the research design. The sample size can influence the test methods used. In particular, RMSEA and TLI have a significant risk of first- and second-type errors when the sample consists of less than 250 respondents (52). Further, the lack of representativeness of the sample limits the study results regarding their transferability to the target group. Moreover, LPA is a statistical estimation, so the results might suffer from biases that are due to, for example, statistical outliers. However, theoretical plausibility provides grounds on which to assume that the found profiles represent different conceptualizations of resilience. Moreover, our study is based on cross-sectional data, so we can make no assessment about causality. Therefore, our interpretations are based on theoretical assumptions about the direction of relationships. Longitudinal studies and studies with larger samples are needed to address these limitations.

Another limitation results from the measures used in our study, as scholars often criticize the use of coping checklists (42, 74) because these checklists do not cover situational factors and the unique actions and thoughts that go along with coping. Further, not all coping strategies are part of coping checklists. Moreover, the Brief Resilience Scale must be used with caution for our analysis because it is based on the definition of “regeneration.” Therefore, it is no wonder that regeneration is found to occur extensively in the “social-active” profile, which has a similar definition. Therefore, we suggests that future studies measure the three conceptualizations using measures for resistance, regeneration and reconfiguration.

Our study leads to several suggestions for future research. To understand the functioning of resilience and find a proper definition, empirical analyses must consider the combination of resilience and coping. Our results suggest not only that functional coping goes along with resilience but also that some individuals do not need to expend excessive effort on coping to withstand adversity. Therefore, besides considering coping and resilience, the role of appraisal might be relevant to (some kinds of) resilience and should be taken into account.

The typology provided here—the four profiles—requires investigation in other settings. It should be validated in a larger sample and using other target groups to determine its utility. Future research must also identify more explanatory factors that can play a role in the profiles. The literature has delivered such possible explanatory factors as optimism, self-efficacy, and awareness (33) that can be critical to the ability to build resilience, and these should be considered in similar future studies. In addition, dependent variables other than irritation may be fruitful targets of investigation.

With regard to the turnover intentions of nurses, our study creates some new insights into coping and resilience that influence nurses' commitment to their jobs, their organizations, and their colleagues. Given the urgent need for healthcare workers, further research on the factors (e.g., income, working conditions) that influence nurses' commitment is necessary.

## Conclusion

All in all, our results explain four heterogeneous ways to deal with stress and demonstrate that these four ways are associated with different stress- and resilience-related variables. Our results underscore the urgency of clarifying the definition of resilience. We do not take a position on whether a definition or a profile represents resilience best, but provide input for an ongoing discussion. The analysis shows that the three prevailing understandings of resilience can be found in profiles, but also that future scientific debate requires more precision in defining individual resilience. The study contributes to sharpening the definition of resilience and understanding the association between coping and resilience. It also contributes to research on the individual resilience of nurses.

## Data Availability

The raw data supporting the conclusions of this article will be made available by the authors, without undue reservation.
